# Methyl Orange Degradation Using Ag-Doped TiO_2_, H_2_O_2_, and Hydrodynamic Cavitation

**DOI:** 10.1021/acsomega.5c00034

**Published:** 2025-05-19

**Authors:** Ryma Merdoud, Farid Aoudjit, Lotfi Mouni, Vaishnavi Honavar, Roja Parvizi Moghadam, Manoj Palabathuni, Vivek V. Ranade

**Affiliations:** † Laboratoire Matériaux et Développement Durable, Faculté des Sciences et Sciences Appliqués, 389770 Université de Bouira, Bouira 10000, Algeria; ‡ Department of Chemical Sciences and Bernal Institute, 8808 University of Limerick, Ireland V94 T9PX, Ireland; § Laboratoire de Gestion et Valorisation des Ressources Naturelles et Assurance Qualité, Faculté SNVST, 389770 Université de Bouira, Bouira 10000, Algeria

## Abstract

This study investigates the photocatalytic degradation of Methyl Orange (MO) using doped photocatalysts, specifically Ag-TiO_2_ synthesized via a novel solid-state method, with varying silver concentrations (0%, 0.5%, 1%, 1.5%, and 2.5% w/w relative to TiO_2_) under different UV light intensities (60 and 200 W). The photocatalysts were characterized using XRD, SEM-EDS, and BET. The optimal performance was observed with a 0.5% Ag-TiO_2_ concentration, achieving a degradation efficiency of 59% under 200 W UV light over 180 min of treatment. The effect of photocatalyst loading was then optimized, followed by an investigation of the synergistic effects of photocatalysis (PC) coupled with hydrogen peroxide (H_2_O_2_). The highest degradation efficiency of 94% was achieved at 0.01% v/v H_2_O_2_ with a synergistic coefficient of 24, within 60 min. Further enhancement was observed when combining PC, H_2_O_2_, and hydrodynamic cavitation (HC), achieving complete degradation of MO in just 3 min (1.5 passes) with a high synergistic coefficient of 42. The degradation process was represented as pseudo-first-order kinetics for PC alone and combined with H_2_O_2_, and a per-pass degradation model for HC. The impact of various scavengers on the photocatalytic process was examined, highlighting the crucial roles of hydroxyl radicals (•OH) and photogenerated holes (h^+^) in the degradation mechanism. The influence of anions and the water matrix on the reactive oxygen species (ROS) generation and efficiency, as well as the environmental fate of Ag-TiO_2_ catalysts, is also discussed. This research underscores the importance of optimizing doped photocatalyst composition and operational conditions to maximize pollutant degradation efficiency, demonstrating significant advancements in advanced oxidation processes through synergy.

## Graphical abstract

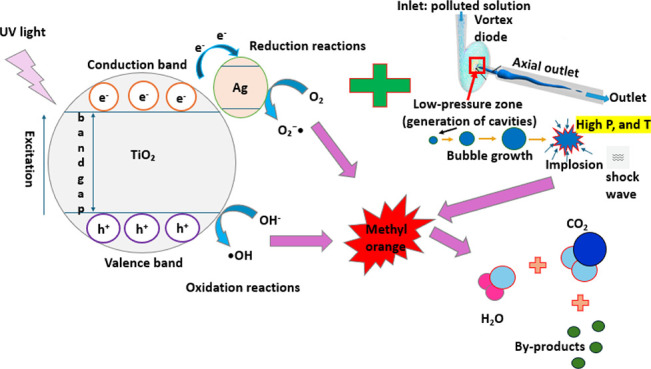

## 1 Introduction

The presence of synthetic dyes in water bodies is a major environmental concern, as it degrades water quality and endangers aquatic life.^1^ This issue is exacerbated by the widespread use of these dyes in various industries, including textiles, food, and pharmaceuticals.^2^ These dyes are often toxic, carcinogenic, and resistant to natural biodegradation processes, posing significant risks to aquatic ecosystems and human health^3–5^ Conventional wastewater treatment methods often fail to completely degrade or mineralize these dyes, allowing them to remain completely or partially in treated water.^6^ Therefore, effective degradation methods are essential to mitigate these environmental impacts.^7^ In this work, we aim to develop such effective degradation methods. Methyl orange (MO) is used in this work as a model pollutant for evaluating the efficiency of new treatment technologies. MO’s complex aromatic structure makes it resistant to conventional degradation methods, offering a reliable benchmark for advanced techniques. Furthermore, MO represents a broader class of azo dyes that are commonly found in industrial effluents, making research on its degradation highly relevant for real-world applications.^8^

Various traditional methods have been employed for the degradation of MO including adsorption,^9^ filtration,^10^ coagulation,^11^ precipitation,^12^ and biological methods.^13^ All of these traditional treatment technologies for removing organic dyes face limitations such as high energy and chemical demands, elevated operating costs, sludge production, and the release of toxic gases, reflecting the need for effective treatment technologies.^14^ Among these, AOPs have gained significant attention due to their ability to generate highly reactive species that can effectively degrade complex organic pollutants.^15^ Photocatalysis, particularly using titanium dioxide (TiO_2_), has been extensively studied for MO degradation.^16^ This method leverages the photocatalytic properties of TiO_2_ under UV light to produce reactive oxygen species that break down MO molecules. However, the efficiency of PC can be limited by the rapid recombination of electron–hole pairs, which reduces the availability of reactive species.^17^ Doped photocatalysts have emerged as a promising approach to enhance the efficiency of photocatalytic degradation of organic pollutants like MO.^18^

Doping introduces foreign atoms into the crystal lattice of the photocatalyst, improving charge separation by reducing electron–hole recombination and thereby increasing the generation of reactive species essential for degradation. Furthermore, doping can alter the surface properties of photocatalysts, such as modifying surface area, surface energy, or the availability and arrangement of active sites. All of these factors may influence the interaction of the catalyst with dye molecules. This combination of effects significantly boosts photocatalytic performance. Silver (Ag) doping is commonly used to enhance the photocatalytic performance of TiO_2_. Its effectiveness is particularly notable due to the plasmonic effect, which enhances the local electromagnetic field, thus increasing photocatalytic activity. Ag acts as an efficient electron trap, improving charge separation and reducing electron–hole recombination, which enhances the generation of reactive species crucial for the degradation of organic pollutants like MO. Studies have shown that Ag doping significantly improves the photocatalytic efficiency of TiO_2_ in degrading MO under UV light. Moreover, Ag doping has been shown to shift the TiO_2_ photocatalyst’s absorption toward the visible spectrum, allowing for more effective PC under solar light. Nagaraj et al.^19^ demonstrated that incorporating Ag significantly enhances the photocatalytic activity of TiO_2_ under solar light, by reducing its bandgap energy, achieving 100% methylene blue removal within 30 min. Polycationic selenides (PCS) have also demonstrated high photocatalytic efficiency under solar light. Nawaz et al.^20^ reported that 99.47% degradation of Eosin and 99.31% of Crystal Violet (CV) were achieved, under solar light, using PCS. Using response surface methodology, their study identified a quadratic polynomial model as the best fit, with a high *R* ^2^ (0.9994) and an adjusted *R* ^2^ (1.0), demonstrating its accuracy in predicting PCS photocatalytic performance.

Each photocatalyst has distinct advantages. Ag-TiO_2_ is particularly notable for its chemical stability, recyclability, and sustained photocatalytic performance^21,22^ It maintains high efficiency over multiple cycles without significant activity loss.^23,24^ Recent studies have demonstrated that the photocatalytic efficiency remains higher than 85% after five consecutive cycles, with only negligible silver leaching detected.^25,26^ In contrast, PCS demonstrates strong visible light activity but are prone to self-oxidation, affecting their long-term stability.^20^ Given its durability and consistent performance, Ag-TiO_2_ remains a highly attractive option for photocatalytic applications,^25^ reinforcing the importance of Ag-doped materials in environmental remediation research.^27^ Table 1 summarizes recent studies investigating the use of Ag-doped photocatalysts for the degradation of MO.

**Table 1:** Recent Studies on Ag-Doped Photocatalysts for the Degradation of MO

Reference	Photocatalyst	Dye	Degradation Efficiency	Conditions	Key Findings
^28^	Ag-ZnO	MO, RhB	MO: 99.3%, RhB: 99.7%	UV light, 35 min	Superior photoactivity and recyclability
^29^	Ag-ZnO nanofibers	MB, RhB, MO	MB: ∼100% (75 min), RhB: ∼66% (120 min), MO: ∼51% (120 min)	UV light	Ag doping significantly improved degradation rates
^30^	Ag-TiO_2_	MO	MO: 95%	UV light, 120 min	Higher efficiency by using Ag-TiO_2_ compared to pure TiO_2_. Pure TiO_2_ data: MO: ∼50%.
^31^	Ag-TiO_2_	MO	MO: ∼100%	UV light, 55 min	Complete degradation by using Ag-TiO_2_ compared to pure TiO_2_. Pure TiO_2_ data: MO: ∼60%.
^32^	Ag-TiO_2_	MO	MO: 95%	UV light, 60 min	Ag-TiO_2_ achieved 95% degradation, while pure TiO_2_ gave about 50%

To further enhance the photocatalytic degradation of pollutants, hydrogen peroxide (H_2_O_2_) is frequently used in photocatalytic systems due to its ability to generate highly reactive hydroxyl radicals (•OH) when exposed to UV light in the presence of the photocatalyst. These radicals are highly effective in breaking down complex pollutants like dyes. Previous studies have demonstrated the benefits of combining H_2_O_2_ with photocatalysts. For example, Shahzad et al.,^33^ demonstrated enhanced degradation of Orange 16 reactive dye using H_2_O_2_-activated ZnO-Bi_2_O_3_ heterostructured composites under UV light. Similarly, Wang et al.,^34^ found that a synergistic Ag/g–C_3_N_4_ and H_2_O_2_ system significantly improved the photocatalytic degradation of azo dyes under UV light. These studies highlight the effectiveness of combining H_2_O_2_ with PC to enhance the degradation of various pollutants, maintaining an oxidative environment necessary for continuous pollutant degradation.

Recently, alternative oxidants in AOPs have gained attention, with sodium percarbonate (SPC) and periodates emerging as promising substitutes for H_2_O_2_. Notably, SPC generates hydroxyl radicals (•OH) upon activation, similar to H_2_O_2_. For instance, Fedorov et al.^35^ demonstrated its effectiveness in degrading 1,4-dioxane when combined with ozone in a hybrid HC system, achieving over 99% degradation efficiency. Similarly, Fan et al.^36^ reported that SPC activated with cobalt oxide (CoO) achieved nearly 94% Reactive Blue 19 dye degradation in just 30 min. In addition to SPC, periodates, have also, gained attention in photocatalytic systems. For example, Chamekh et al.^37^ showed that complete degradation of Orange G dye using UV/TiO_2_/periodate process was achieved within 10 min, highlighting its effectiveness in wastewater treatment. Despite these promising alternatives, H_2_O_2_ remains a widely used and well-established oxidant in AOPs due to its strong oxidative potential, ease of handling, and ability to generate hydroxyl radicals efficiently under various activation methods, including UV, transition metals, and ozone. Moreover, H_2_O_2_ has been extensively studied and applied in wastewater treatment, demonstrating high efficiency in degrading a wide range of organic pollutants, including dyes and pharmaceuticals, making it a reliable and proven choice in AOPs.^38,39^

In addition, HC has proven to be an effective method for enhancing the degradation of organic pollutants, including dyes, in wastewater treatment. HC generates high-temperature and high-pressure conditions within the liquid, producing microbubbles that collapse violently, creating reactive radicals such as hydroxyl radicals (•OH).^40^ These highly reactive species can effectively degrade pollutants, complementing other advanced oxidation processes (AOPs).^41^ Recent studies have explored HC as a promising technique for wastewater treatment. For example, Cao et al.^42^ reported the effectiveness of the combined process of HC/H_2_O_2_/vitamin C, achieving an 87.8% degradation rate for methylene blue (MB) under optimized conditions with a synergy index of 1.61. Merdoud et al.^43^ also highlighted the synergistic effect of combining HC with AOPs such as PC and H_2_O_2_ for optimal pollutant degradation efficiency. Related studies have also demonstrated the effectiveness of combining cavitation with PC, for degrading complex dye pollutants like Rhodamine dyes. Mohod et al.^44^ critically reviewed this approach, showing that the Rhodamine achieved near-complete mineralization under optimized conditions. This synergy highlights the potential of hybrid AOPs for efficiently treating recalcitrant dyes in wastewater. Several types of cavitation devices are used for HC, including orifice-based,^45^ venturi-based,^46^ and vortex-based devices.^43^ Vortex-based cavitation devices developed by Ranade and coworkers,^47,48^ in particular, have been highlighted for their ability to generate more uniform cavitation fields, making them more efficient for large-scale applications.

The present work investigates the hybrid technology of PC, H_2_O_2_, and HC, aiming to explore their synergistic effects for enhanced degradation of organic dyes, particularly MO in wastewater treatment. The rationale for hybrid technologies lies in the complementary nature of each AOP. PC generates reactive oxygen species under UV light, initiating the degradation process. H_2_O_2_ further enhances this by contributing to the generation of hydroxyl radicals, amplifying oxidative degradation. HC complements these mechanisms by producing additional radicals through bubble collapse, creating a synergistic effect that significantly improves pollutant removal efficiency. This work presents a novel approach to synthesizing Ag-doped TiO_2_ photocatalysts using a solid-state synthesis method, a technique that has not been previously applied to this specific complex. Unlike conventional sol–gel or hydrothermal methods, which typically require solvents and multiple processing steps, solid-state synthesis provides a simpler, solvent-free, and potentially more scalable route for Ag-TiO_2_ fabrication. This method offers enhanced control over phase purity and structural stability, making it a promising alternative for photocatalyst development. Moreover, the integration of this photocatalyst with vortex-based HC represents a pioneering advancement, as no prior studies have explored this synergy for wastewater treatment. To the best of our knowledge, this integrated approach has not been previously reported for the degradation of methyl orange or other pollutants. The combination of Ag doping, which improves charge separation, with the intensified mass transfer and radical generation effects of HC, leads to a highly efficient and scalable system for dye degradation. These findings not only introduce an original synthesis route for Ag-TiO_2_ but also demonstrate the potential of the hybrid system, combining Ag-doped TiO_2_ with vortex-based HC establishing a foundation for industrial-scale applications, addressing both efficiency and feasibility in real-world wastewater treatment scenarios.

## 2 Experimental Section

### 2.1 Materials

Methyl orange (molecular weight: 327.334 g/mol; molecular formula: C_14_H_14_N_3_NaO_3_S) was purchased from Thermo Fisher Scientific. Sulfuric acid (Sigma-Aldrich, 98%) was used for adjusting the pH of the solution. Hydrogen peroxide (H_2_O_2_, Thermo Fisher Scientific, 30% v/v) was employed to assess its enhancement of the process oxidant capacity. Titanium­(IV) oxide nanopowder (Sigma-Aldrich, ≥99.5%, trace metals basis) was used as the photocatalyst. Silver­(I) oxide (Sigma-Aldrich, 99%) was used as a dopant for the synthesis. Isopropanol (Sigma-Aldrich, 99.5%), methanol (Sigma-Aldrich, 99.9%), EDTA (Sigma-Aldrich, 99.4–100.6%, powder), and potassium iodide (Sigma-Aldrich, 99%) were used as radical scavengers. All purchased chemicals were applied as received.

### 2.2 Experimental Setup

The experimental setup consists of a lab-scale photoreactor designed to study PC both independently and in combination with HC represented schematically in Figure 1. The photographs of the set-ups are depicted as Figures S1 and S2. For the photocatalytic processes, a 60 W, 395 nm UV light LED, and a 200 W UV light LED were used as light sources. These lamps were mounted horizontally, one at a time, at the top of an aluminum photoreactor with a volume of 64,343 cm^3^ (37 cm height × 37 cm length × 47 cm width), positioned 21 cm above the surface of the liquid. The design of the photoreactor ensures uniform light distribution over the liquid surface, promoting optimal photocatalytic efficiency. During the experiments, a 200 mL solution of MO was placed in a glass beaker and magnetic stirred at 200 rpm to ensure uniform catalyst suspension and avoid settling. The hybrid system consists of a holding tank with a maximum capacity of 2.5 L. The liquid is pumped from this tank using a centrifugal pump with a power rating of 0.75 kW, featuring integrated manual pressure drop control to ensure consistent liquid circulation through the pipe to a vortex-based cavitation device with a throat diameter of 3 mm. The cavitation device, made of aluminum, is based on the design proposed by Ranade et al.^49^ ensuring effective cavitation bubble generation. The temperature of the holding tank was monitored but not controlled during the experiment.

**Figure 1:**
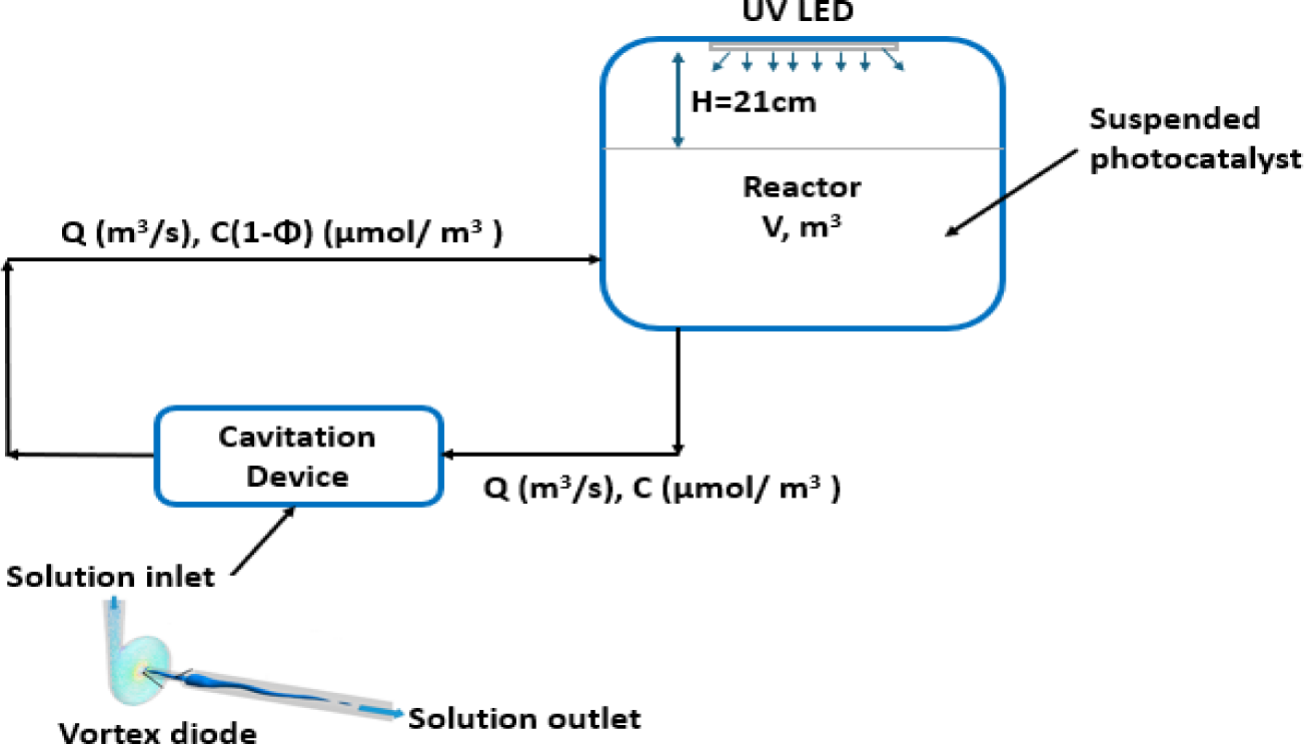
Schematic of experimental setup.

### 2.3 Preparation and Characterization of Ag-TiO_2_ NPs

The Ag-doped TiO_2_ photocatalysts were synthesized using a solid-state method. Initially, 6 g of powdered TiO_2_ was placed in a mortar. Ag_2_O was added in varying concentrations (0%, 0.5%, 1%, 1.5%, and 2.5% w/w relative to TiO_2_). The mixture underwent mechanical grinding with a pestle, employing alternating circular motions and pressing, for 1 h to ensure thorough homogenization. Subsequently, the resultant powder was sieved through a 40 μm mesh to eliminate agglomeration and achieve uniform particle size distribution. The powders were then subjected to calcination at 600 °C for 2 h to facilitate the formation of Ag-TiO_2_ photocatalysts. The final products were stored in dark glass containers, which were tightly sealed to maintain their integrity and protect them from light, oxygen, and contamination. Photographs of the process are depicted in Figures S3 and S4.

The synthesized photocatalytic materials were characterized using various techniques. X-ray diffraction (XRD) patterns were obtained using a PANalytical Empyrean diffractometer, produced by Malvern PANalytical, with Cu Kα radiation at a scan rate of 0.1° 2θ per second, operating at 45 kV and 40 mA. The limit of detection (LOD) ranges from 0.1% to 1% by weight for crystalline phases, and the limit of quantification (LOQ) is around 1% to 5% by weight. Scanning electron microscopy (SEM) images were captured using a Hitachi SU-70 microscope, produced by Hitachi High-Tech, at 5 kV, featuring a BSE detector with 3 nm resolution, and equipped with EDS for elemental analysis and mapping. The LOD is typically in the range of 0.1–1% by weight for most elements, while the LOQ is around 1–10% by weight. Brunauer–Emmett–Teller (BET) analysis was conducted using a TriStar II Plus (Tristar 3030) analyzer with pure liquid nitrogen at 77.3 K. The samples were degassed at 100 °C for 10 h to accurately determine the photocatalyst’s specific surface area. The LOD is around 0.01 m^2^/g, while the LOQ is around 0.1 m^2^/g.

For ensuring the reliability and reproducibility of the experimental results, standard quality control measures were implemented. For XRD analysis, an internal silicon standard was used to verify instrument calibration and minimize peak shifts. SEM-EDS measurements were conducted with multiple imaging conditions to confirm structural and compositional consistency, and a reference material was analyzed to validate the EDS quantification accuracy. For BET analysis, the instrument was regularly calibrated, and multiple adsorption–desorption cycles were performed to confirm data stability. Additionally, all experiments were conducted in a controlled environment to minimize contamination, and duplicate measurements were taken to assess reproducibility.

### 2.4 Experimental Methodology

The present work is divided into the following three sets of experiments:

#### 2.4.1 Set 1: PC Alone

In this set of experiments, all tests were conducted in the same photoreactor with 200 mL of MO solution, with concentrations ranging from 14 to 15 ppm and a pH adjusted using H_2_SO_4_ to approximately 3, as per previous studies.^50,51^ A loading of 1 g/L of photocatalysts was used, and an adsorption time of 60 min was established under magnetic stirring at 200 rpm. Following this, the PC was performed under UV LED irradiation for 180 min. Both TiO_2_ and Ag-doped TiO_2_ were subjected to UV light irradiation using two separate UV LED lamps (60 and 200 W). Ag-TiO_2_ was tested at different doping percentages (0%, 0.5%, 1%, 1.5%, and 2.5% Ag-TiO_2_ w/w relative to TiO_2_) to determine the optimal light intensity and the most effective photocatalyst for MO degradation. Samples (2 mL) were collected every 30 min for analysis. Using the optimal photocatalyst type and light intensity, the effect of photocatalyst loading was also investigated. Three loadings were tested: 0.5 g/L, 1 g/L, and 1.5 g/L.

#### 2.4.2 Set 2: PC and H_2_O_2_

After determining the optimal conditions of photocatalyst type, light intensity, and photocatalyst loading in the first set of experiments, the synergistic effect of PC in combination with varying concentrations of H_2_O_2_ (30% v/v) was studied. The concentrations tested were 0%, 0.001%, 0.005%, and 0.01% v/v, with 2 mL of samples taken every 10 min over 60 min after switching on the lamp.

#### 2.4.3 Set 3: PC, H_2_O_2_, and HC

Finally, MO degradation was investigated using a hybrid system consisting of PC and H_2_O_2_, combined with HC in a 2.5 L MO solution under the optimal conditions determined in the first and second sets of experiments. The vortex diode (3 mm) was used at the optimal inlet pressure of 150 kPa, which was determined in our previous work.^43^ The experiment was conducted for 60 min, with the MO solution placed in the dark and stirred at 200 rpm for 60 min to reach adsorption and desorption equilibrium before turning on the light and initiating the PC process. The effect of different radical scavengers was studied using the optimized conditions for photocatalyst type, light intensity, and photocatalyst loading. Throughout all experiments, the photocatalyst was separated from the solution using a PTFE filter (0.2 μm), and the solution was analyzed to determine the dye concentration at λ_max_ 470 nm. The pH of the solutions was monitored and maintained at approximately 2.8–3. All dye degradation experiments were performed in triplicate, with experimental errors found to be less than 5%.

### 2.5 Analysis and Processing of Data

The samples collected were analyzed using a UV–Vis spectrophotometer (Shimadzu UV1800) to track changes in absorbance over time at a specific wavelength, which varied with the pH of the medium. At pH = 3, the maximum absorbance peak of the MO solution was observed at 470 nm (Figure S5). The MO solution concentrations were determined using a calibration curve, and all concentration values obtained from UV measurements were considered accurate for subsequent calculations. The rate of MO removal was calculated using the following equation:^52^

1 $$\%\mathrm{MO removal}=\frac{C_{0}-C}{C_{0}}\times 100$$

Where *C*_0_ is the initial concentration of MO, and *C* [ppm] is the concentration at time *t*.

The degradation kinetics of MO were modeled using different approaches depending on the method applied. For PC alone, the experimental data was interpreted using a first-order kinetics, represented by eq 2:^53^

2 $$C=C_{0}e^{-kt}$$

where *k* [min^–1^] is the first-order rate constant of the reaction and *t* is time [min].

In our previous work, we discussed that pseudo-first-order kinetics have been commonly employed to describe the pollutant degradation via HC.^54–56^ Alternatively, the per-pass model proposed by Ranade et al.,^57^ can also be applied. In this work, the overall behavior of a typical cavitation-based water-treatment setup was modeled using both these approaches as

3 $$V\frac{dC}{dt}=Q\Phi C,\mathrm{or},\frac{dC}{dt}=-k_{\mathrm{eff}}C$$

where *C* [ppm] represents the concentration of pollutants, *V* [L] denotes the working volume (including the holding tank and the volume of piping with the pump), and *Q* [L/min] is the flow rate through the cavitation device. The apparent degradation rate constant is *k*_eff_ [min^–1^], and Φ [−] is the per-pass degradation factor. Although it is possible to account for temperature effects by considering activation energy, this study does not include such considerations. Instead, we report the effective values of the per-pass factor Φ or the effective rate constant *k*_eff_, estimated over the temperature range used in the experiments.

## 3 Results and Discussion

### 3.1 Characterization of TiO_2_-Based Photocatalysts

#### 3.1.1 XRD

Figure 2 represents the XRD pattern of pureTiO_2_ and Ag-TiO_2_ at varying doping levels (0.5%, 1%, 1.5%, and 2.5%). It reveals the dominant anatase phase with characteristic peaks at 25.3°, 37.8°, 48.0°, 53.9°, and 55.1° (2θ), corresponding to the (101), (004), (200), (105), and (211) planes, respectively. The stability of these peaks across all doping levels indicates that the anatase structure is preserved despite incorporating silver.^58^ Additionally, a minor peak at 27.4° (2θ), associated with the rutile phase (110) plane, is present in all samples, but it remains weak, confirming that anatase is the predominant phase.^59^ Doping with 0.5% Ag notably enhances the intensity of the anatase peak at 25.3°, reflecting improved crystallinity and a reduction in structural imperfections.^60^

**Figure 2:**
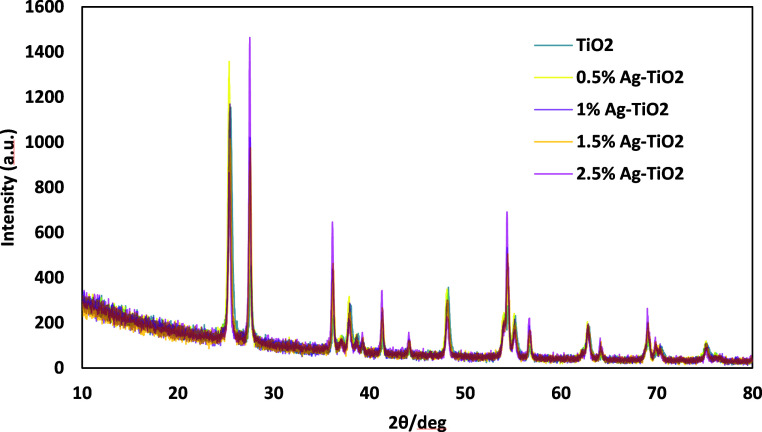
XRD patterns of undoped TiO_2_, and Ag-TiO_2_ photocatalysts at different doping levels (0.5%, 1%, 1.5%, and 2.5% w/w relative to TiO_2_).

This enhancement suggests that a lower concentration of silver stabilizes the anatase phase by strengthening the lattice structure and minimizing defects. At this optimal concentration, the incorporation of silver fills oxygen vacancies without significantly distorting the lattice, thereby improving crystallinity.^61^ Additionally, at 0.5%, the dopant level remains low enough to avoid triggering the anatase-to-rutile transformation, allowing the anatase phase to dominate which is critical for achieving optimal photocatalytic performance. At higher doping levels, such as 1%, 1.5%, and 2.5%, a marked increase in the intensity of the rutile peak at 27.4° (2θ), corresponding to the (110) plane, is observed. This phenomenon may be due to the cumulative lattice distortions induced by the incorporation of larger amounts of silver atoms.^18^ Higher dopant concentrations may introduce significant stress within the TiO_2_ matrix, destabilizing the anatase phase and lowering the energy barrier for the anatase-to-rutile transformation.^62^ Furthermore, the increased silver content may generate more structural defects and oxygen vacancies, which act as nucleation sites for the growth of rutile crystallites.^63^ Thus, while lower silver concentrations stabilize anatase, higher concentrations destabilize it and promote the rutile phase.

Despite the structural modifications, no distinct peaks corresponding to silver or silver oxide phases are detected. This absence may indicate that silver is either highly dispersed within the TiO_2_ lattice or present in amounts below the detection sensitivity of XRD, confirming successful doping without the formation of segregated silver phases.^64^ The preservation of the anatase structure, even at higher doping levels, demonstrates the robustness of the TiO_2_ lattice and the effective incorporation of silver.^65^

#### 3.1.2 SEM-EDS

Figure 3 represents the SEM-EDS mapping and spectra of undoped TiO_2_, and Ag-TiO_2_ with different proportions of Ag NPs (0.5%, 1%, 1.5%, and 2.5% w/w relative to TiO_2_), highlighting the elemental distribution, structural features, and morphological characteristics of each sample. For the pure TiO_2_ sample, the mapping illustrates a uniform titanium distribution of particles across the surface, as depicted by the green spots. The EDS spectrum further confirms the high purity and structural homogeneity of TiO_2_, showing distinct peaks for titanium and oxygen. In the 0.5% Ag-TiO_2_ sample, the SEM images reveal the initial incorporation of silver into the TiO_2_ matrix, with silver particles appearing as small, evenly distributed red spots. The EDS spectrum shows the presence of silver alongside titanium and oxygen, with a low-intensity Ag peak, reflecting successful doping at a low concentration.^66^ For the 1%, 1.5%, and 2.5% Ag-TiO_2_ samples, the SEM images show an increasing density of red spots, indicating higher silver content. At 1%, the silver particles are well-dispersed, enhancing photocatalytic potential without significant aggregation. At 1.5%, some clustering of silver particles begins to appear, which could slightly reduce the uniformity of the distribution. At 2.5%, the images reveal clear evidence of particle agglomeration, which might hinder photocatalytic efficiency due to reduced active surface area. The EDS spectra for these samples show progressively intensified Ag peaks, confirming the higher silver concentrations.

**Figure 3:**
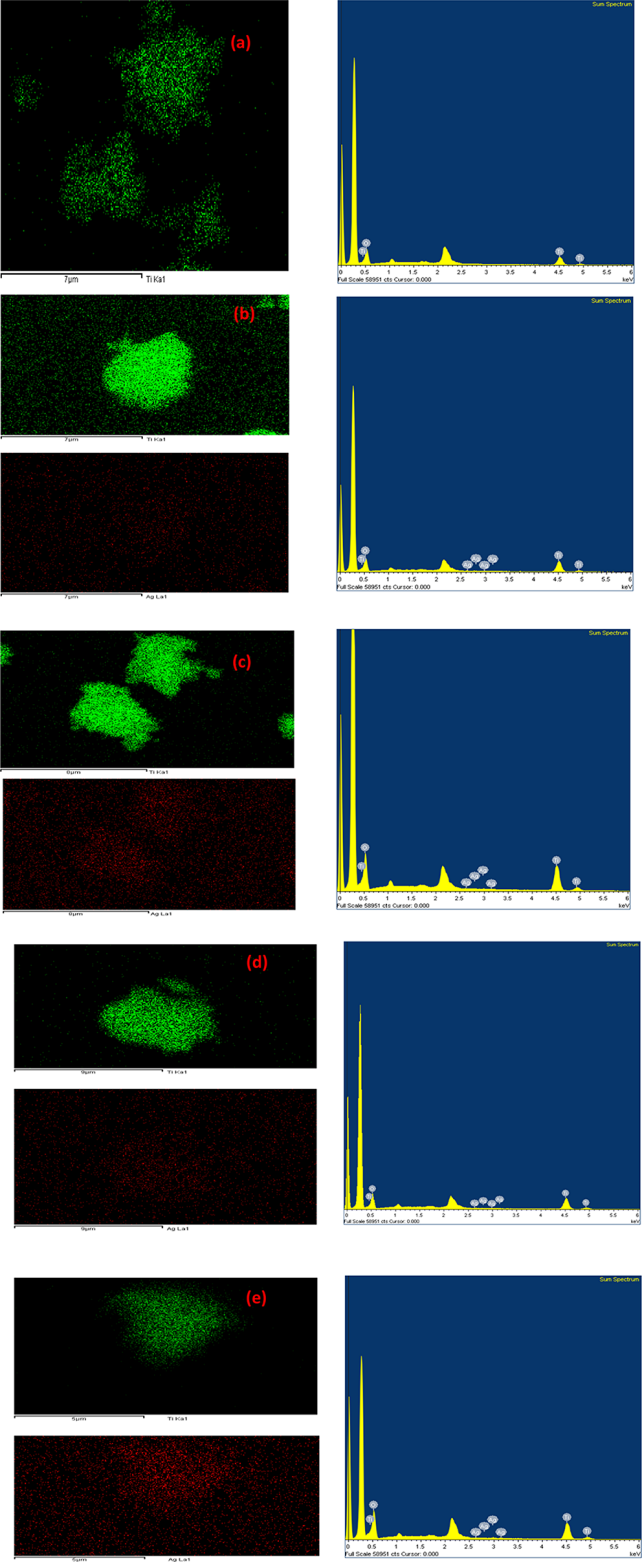
SEM-EDS mapping analysis and spectra of (a) TiO_2_, (b) 0.5% Ag-TiO_2_, (c) 1% Ag-TiO_2_, (d) 1.5% Ag-TiO_2_, (e) 2.5% Ag-TiO_2_ photocatalysts.

#### 3.1.3 BET Analysis

The BET analysis reveals significant trends in the textural properties of TiO_2_ and Ag-doped TiO_2_ photocatalysts, which directly correlate with their photocatalytic activity. The pore size of undoped TiO_2_ was 118.44 Å, with a surface area of 30.44 m^2^/g, providing a baseline mesoporous structure ideal for PC. Upon doping with 0.5% Ag, the pore size (116.95 Å) and surface area (28.86 m^2^/g) remain relatively unchanged, maintaining sufficient active sites and reactant accessibility while introducing the plasmonic effect of silver, which enhances light absorption and minimizes charge recombination. This balance results in 0.5% Ag-TiO_2_ being the most efficient catalyst for MO degradation. At higher doping levels, structural modifications become more pronounced. The 1% Ag-TiO_2_ exhibits a slight increase in pore size (121.37 Å) but a decrease in surface area (23.79 m^2^/g), suggesting agglomeration of silver particles that begin to obstruct active surface regions. Further increases in Ag content (1.5% and 2.5%) result in significant reductions in both pore size (105.85 Å and 63.66 Å, respectively) and surface area (22.47 m^2^/g and 15.22 m^2^/g). These reductions indicate excessive silver deposition blocking fine capillaries, diminishing the accessible catalytic surface, and potentially introducing recombination centers for electron–hole pairs, thus undermining photocatalytic efficiency.

Overall, the BET analysis demonstrates that low silver doping levels (0.5%) optimize the synergy between enhanced light absorption and maintained structural properties, higher silver concentrations disrupt this balance, leading to diminished photocatalytic performance. Table 2 summarizes the BET analysis of TiO_2_ and Ag-doped TiO_2_ photocatalysts. For comparison, Sobana et al.,^67^ reported that the surface area decreased from 21.53 m^2^/g (pure TiO_2_) to 17.78 m^2^/g with 1.5% Ag doping, and further to 12.39 m^2^/g with 2% Ag doping. Similarly, Chuang et al.,^68^ found that Ag doping reduced TiO_2_’s surface area from 196.8 m^2^/g to 117.8 m^2^/g. These findings align with our observations that silver doping generally leads to a slight decrease in surface area, which can impact photocatalytic efficiency.

**Table 2:** BET Surface Area and Pore Size Analysis of TiO_2_ and Ag-Doped TiO_2_ Photocatalysts

Photocatalyst	Pore size (Å)	BET surface area (m^2^/g)
0% Ag-TiO_2_	118.44	30.44
0.5% Ag-TiO_2_	116.95	28.86
1% Ag-TiO_2_	121.37	23.79
1.5% Ag-TiO_2_	105.85	22.47
2.5% Ag-TiO_2_	63.66	15.22

### 3.2 Influence of Silver Concentrations and Different Lamp Intensities on MO Degradation

This study investigates the effect of Ag-TiO_2_ photocatalysts with different silver concentrations (0%, 1%, 1.5%, and 2.5% w/w relative to TiO_2_) on MO degradation under different UV LED lamp intensities (60 and 200 W). At 60 W, as shown in Figure 4a, the 0.5% Ag-TiO_2_ exhibited the highest degradation efficiency (24.6%) with a kinetic constant of 0.0016 min^–1^, indicating faster reaction rates, over 180 min of treatment. This enhanced performance is attributed to silver’s role as an electron trap, capturing photogenerated electrons and reducing the recombination of electron–hole pairs, thereby prolonging the lifespan of reactive species responsible for MO degradation. However, as the silver concentration increases, the degradation efficiency decreases. At 1% Ag-TiO_2_, the efficiency drops to 21.7% with a kinetic constant of 0.0014 min^–1^, indicating slower reaction rates, likely due to partial agglomeration of silver particles, which reduces their dispersion and effectiveness. Further increases to 1.5% and 2.5% Ag-TiO_2_ result in even lower efficiencies of 14.3% (k = 0.0008 min^–1^) and 5.3% (k = 0.0003 min^–1^), respectively. This decline is attributed to excessive silver coverage hindering light absorption and blocking active sites on the TiO_2_ surface, as well as promoting recombination of electron–hole pairs. For pure TiO_2_, a degradation efficiency of 19.6% and a kinetic constant of 0.0013 min^–1^ were achieved.

**Figure 4:**
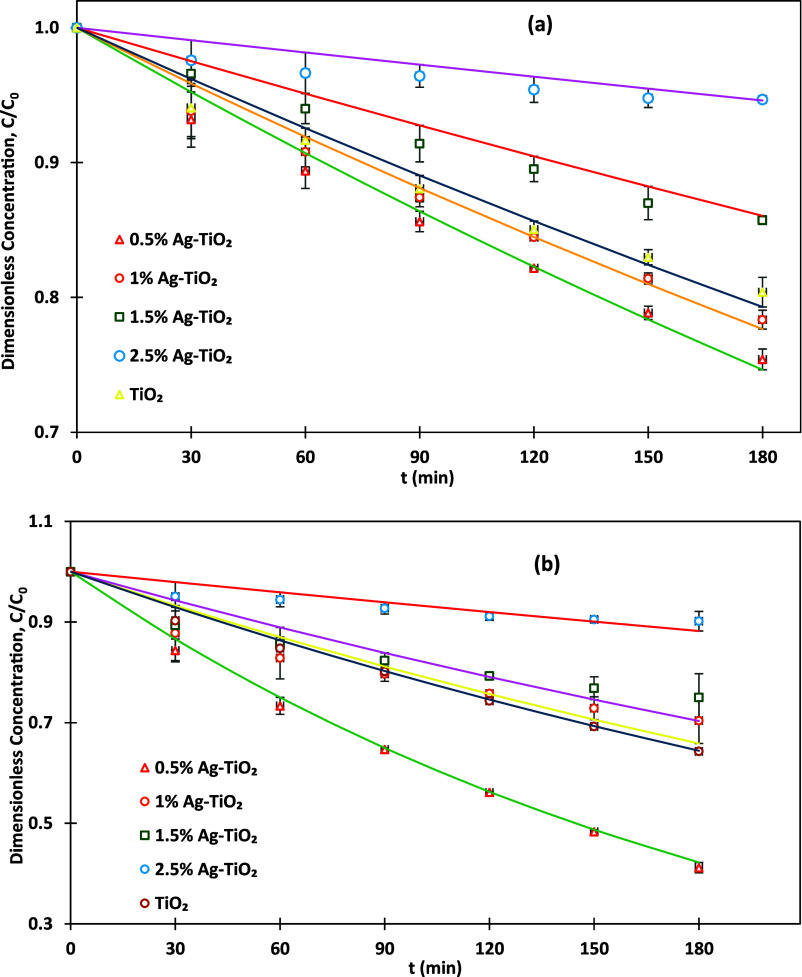
MO dye degradation versus time for different photocatalysts under different UV-irradiation intensities: (a) 60 W and (b) 200 W; photocatalysts dose = 1 g/L, pH = 3, and MO initial concentration = 15 ppm.

In contrast, Figure 4b shows that at a higher lamp intensity of 200 W, the photocatalytic performance significantly improves across all silver concentrations. The 0.5% Ag-TiO_2_ photocatalyst exhibits a dramatic increase in degradation efficiency to 58.8% with a kinetic constant of 0.0048 min^–1^, highlighting the critical role of higher light intensity in enhancing photocatalytic activity. The increased light intensity generates more photogenerated electron–hole pairs, improving the availability of reactive oxygen species required for MO degradation. Even pure TiO_2_ sees a performance boost, achieving a degradation efficiency of 35.7% and a kinetic constant of 0.0024 min^–1^. However, similar to the 60 W intensity, increasing the silver concentration beyond 0.5% leads to diminished performance. At 1% Ag-TiO_2_, the degradation efficiency reduces to 29.6% (*k* = 0.0023 min^–1^), and further declines to 25% (*k* = 0.002 min^–1^) and 9.8% (*k* = 0.0007 min^–1^) at 1.5% and 2.5% Ag-TiO_2_, respectively. The consistent trend of declining efficiency with higher silver concentrations is due to agglomeration, light blocking, and increased recombination of charges. Lines in Figure 4 are as per eq 2 with rate constants listed in Table 3. The kinetic rate constants (*k*) were fitted by minimizing the square of errors between predicted values and experimental data.

**Table 3:** Kinetic Parameters and Degradation Rates at Varying UV Intensities

Photocatalyst	UV intensity [W]	Time of degradation [min]	*k* [min^–1^]	Degradation rate [%]	*R*^2^ [−]
0% Ag-TiO_2_			0.0013	19.6	0.9989
0.5% Ag-TiO_2_			0.0016	24.6	0.9982
1% Ag-TiO_2_	60	180	0.0014	21.7	0.9987
1.5% Ag-TiO_2_			0.0008	14.3	0.9995
2.5% Ag-TiO_2_			0.0003	5.3	0.9999
0% Ag-TiO_2_			0.0024	35.7	0.996
0.5% Ag-TiO_2_			0.0048	58.8	0.9849
1% Ag-TiO_2_	200	180	0.0023	29.6	0.9964
1.5% Ag-TiO_2_			0.0020	25.0	0.9974
2.5% Ag-TiO_2_			0.0007	9.8	0.9997

While higher light intensity (200 W) significantly enhances degradation rates and reaction kinetics, the optimal silver concentration remains at 0.5% Ag-TiO_2_. This concentration balances charge separation and maintains an accessible TiO_2_ surface for photocatalytic reactions. Excessive silver doping, regardless of light intensity, leads to agglomeration and recombination issues that negate the benefits of increased lamp power. Table 3 summarizes the intensity and silver concentration effect on MO degradation.

### 3.3 Effect of Photocatalyst Loading on MO Degradation

Figure 5 represents the influence of different photocatalyst concentrations (0.5 g/L, 1 g/L, and 1.5 g/L) on MO degradation using the optimum photocatalyst (0.5% Ag-TiO_2_) under optimum UV LED light source (200 W). At 0.5 g/L, a degradation efficiency of 25.7% was obtained, with a rate constant (*k*) of 0.0019 min^–1^. The slower degradation rate can be attributed to the insufficient photocatalyst concentration, which provided a limited number of active sites for the photocatalytic reaction. As a result, fewer pollutant molecules interacted with the photocatalyst, leading to lower removal efficiency. At 1 g/L, the degradation efficiency improved significantly to 58.8%, with a higher rate constant (*k*) of 0.0048 min^–1^, indicating optimal conditions. At this loading, the photocatalyst provided an adequate number of active sites and maintained good dispersion in the solution. The higher availability of active sites allowed efficient interaction between the pollutant molecules, the photocatalyst, and the light source, leading to enhanced degradation. However, at 1.5 g/L, the degradation efficiency decreased to 38.2%, with a lower rate constant (*k*) of 0.0028 min^–1^. Despite the higher photocatalyst concentration, the degradation rate dropped due to particle agglomeration, which reduced the effective surface area and the number of accessible active sites. Additionally, the excessive photocatalyst may have blocked light penetration, limiting the activation of photocatalytic sites and thereby reducing efficiency. Thus, the optimal performance was observed at 1 g/L, where the 0.5% Ag-TiO_2_ photocatalyst achieved the best combination of sufficient active sites and efficient light utilization. Lines in Figure 5 are as per eq 2 with rate constants listed in Table 4. Table 4 summarizes the impact of photocatalyst dosages on MO degradation.

**Figure 5:**
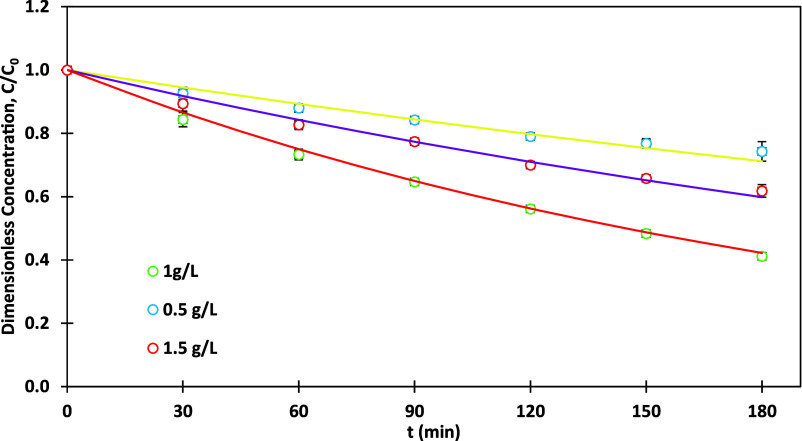
MO dye degradation versus time for different photocatalyst dosages; 0.5% Ag-TiO_2_, 200 W UV-irradiation, pH = 3, and MO initial concentration = 15 ppm.

**Table 4:** MO Degradation, and Rate Constant *k* in Different Photocatalyst Dosages

Photocatalyst dosage [g/L]	Photocatalyst	Time of degradation [min]	Rate constant *k* [min^–1^]	Degradation rate [%]
0.5			0.0019	25.7
1	0.5% Ag-TiO_2_	180	0.0048	58.8
1.5			0.0028	38.2

### 3.4 Synergy Effect of H_2_O_2_ in Photocatalysis

The graphs in Figure 6 explore the synergy effect of H_2_O_2_ in combination with PC for the degradation of MO under 200 W UV light, using a 0.5% Ag-TiO_2_ at 1 g/L photocatalyst concentration. The synergy coefficient (γ_1_) quantifies the enhancement in degradation efficiency due to the combined effect of PC and H_2_O_2_ compared to their individual contributions. It is calculated using the following equation:^69^

4 $$\gamma_{1}=\frac{k_{\mathrm{PC}+H_{2}O_{2}}}{k_{\mathrm{PC}}+k_{H_{2}O_{2}}}$$

**Figure 6:**
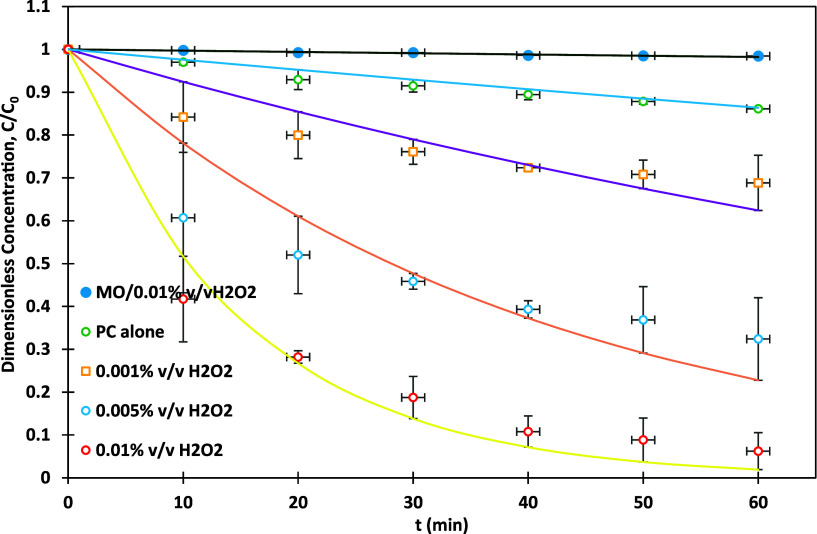
MO dye degradation versus time for different C concentrations; 0.5% Ag-TiO_2_, 200 W UV-irradiation, photocatalyst dosage= 1 g/L, pH = 3, and MO initial concentration = 15 ppm.

Where $k_{\mathrm{PC}+H_{2}O_{2}}$ is the rate constant of PC with H_2_O_2_, *k*_PC_ is the rate constant of PC without H_2_O_2_, and $k_{H_{2}O_{2}}$ is the rate constant of MO with H_2_O_2_ without the photocatalyst.

When photolysis was performed with 0.01% v/v H_2_O_2_ in the absence of the photocatalyst, the degradation efficiency was minimal at 1.6%, with a low-rate constant of 0.003 min^–1^. This indicates that UV light alone, even in the presence of H_2_O_2_, is insufficient to generate a significant number of ROSs such as hydroxyl radicals (•OH), which are key to the oxidative degradation process for effective degradation. The reliance on photolysis without the photocatalytic activation of H_2_O_2_ limits its performance. The PC alone, without the addition of H_2_O_2_, results in a modest degradation efficiency of 13.9% over 60 min of treatment, with a slightly lower rate constant of 0.0024 min^–1^. Here, the activity is driven by the photoinduced electron–hole pairs generated by Ag-TiO_2_ under UV light. However, the absence of H_2_O_2_ means that the production of ROSs is limited, and the recombination of electron–hole pairs further reduces the overall degradation efficiency.

In contrast, the addition of small concentrations of H_2_O_2_ significantly improved the photocatalytic performance. At 0.001% v/v H_2_O_2_, the degradation efficiency has increased to 31.2%, with a rate constant of 0.0079 min^–1^. The presence of H_2_O_2_ enhances ROS production by interacting with photogenerated electrons, forming hydroxyl radicals that contribute to the oxidative degradation of MO molecules. The γ_1_ for this concentration was 2.93. As the concentration of H_2_O_2_ was increased to 0.005% v/v, the degradation efficiency was raised sharply to 67.6%, with a higher rate constant of 0.0247 min^–1^. This improvement reflects the more effective generation of ROSs, as H_2_O_2_ not only supplies hydroxyl radicals but also acts as an electron scavenger, reducing recombination. The γ_1_ for this concentration was 9.15. The highest degradation efficiency of 93.8% was achieved with 0.01% v/v H_2_O_2_, corresponding to a rate constant of 0.066 min^–1^. At this concentration, H_2_O_2_ plays a dual role by both suppressing electron–hole recombination and serving as a continuous source of hydroxyl radicals. The system’s performance peaks under these optimized conditions, demonstrating the powerful synergy effect between H_2_O_2_ and PC. The molar ratio of H_2_O_2_ to MO was calculated and is approximately 228,741. This large excess of H_2_O_2_ compared to MO suggests that H_2_O_2_ has significantly enhanced the degradation of MO by generating more hydroxyl radicals and improving the overall photocatalytic effectiveness. The detailed calculations are provided in Section S1. The γ_1_ for this concentration is 24.44, which has not been previously achieved for MO using Ag-TiO_2_. Lines in Figure 6 are as per eq 2 with rate constants listed in Table 5. Table 5 summarizes the rate constant k, the degradation rate of MO degradation, and the synergy coefficient for different H_2_O_2_ concentrations.

**Table 5:** Synergistic Effect of H_2_O_2_ on Photocatalytic Degradation of Methyl Orange

Operative conditions	Time of degradation [min]	Rate constant *k* [min^–1^]	Degradation rate [%]	Synergy coefficient γ_1_ [−]
PC alone	60	0.0024	13.9	-
MO/0.01% v/v H_2_O_2_ alone	60	0.0003	1.6	-
PC + 0.001% v/v H_2_O_2_	60	0.0079	31.2	2.93
PC + 0.005% v/v H_2_O_2_	60	0.0247	67.6	9.15
PC + 0.01% v/v H_2_O_2_	60	0.0660	93.8	24.44

### 3.5 MO Degradation by PC, H_2_O_2_, and HC Coupling

The study evaluates the efficiency of a hybrid system combining photocatalysis, hydrogen peroxide, and hydrodynamic cavitation, for the degradation of MO. Figure 7 illustrates the remarkable performance of this approach under various operational conditions. Using 0.5% Ag-TiO_2_, PC alone achieved a modest degradation efficiency of 13.9% over 60 min, while HC alone, at optimum pressure of 1.5 bar, resulted in a significantly higher degradation efficiency of 83.3% over the same duration, as investigated in our previous study,^43^ with a *k*_eff_ of 0.0445 (see Section S2 for more details on the calculation of *k*_eff_). At an optimal H_2_O_2_ concentration of 0.01% and in combination with HC, the system achieved complete degradation of MO in just 3 min (1.5 passes), corresponding to a *k*_eff_ of 1.9746 min^–1^. This improvement is attributed to HC, which creates intense turbulence and localized high-energy zones, enhancing the interaction between MO, the photocatalyst, and reactive radicals. The calculated synergy coefficient (γ_2_) for this condition was 42, calculated as per eq 5, indicating a highly efficient interaction between the AOPs.

5 $$\gamma_{2}=\frac{k_{\mathrm{PC}+H_{2}O_{2}+\mathrm{HC}}}{k_{\mathrm{PC}}+k_{H_{2}O_{2}}+k_{\mathrm{HC}}}$$

**Figure 7:**
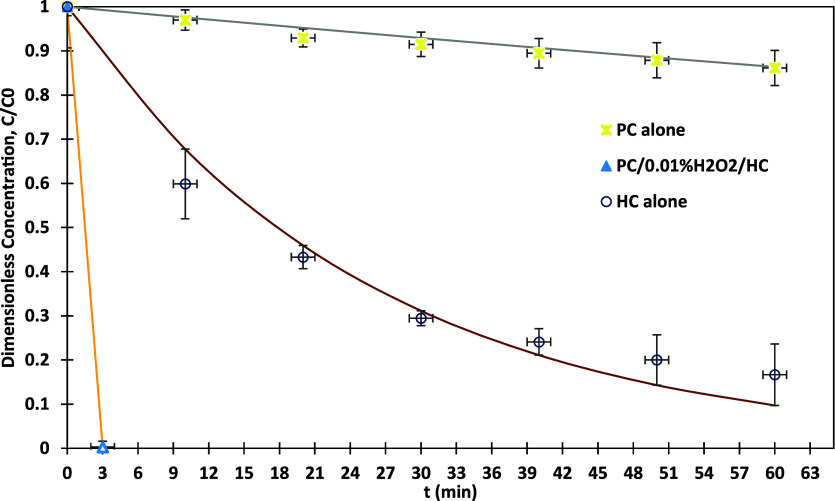
MO dye degradation versus time using photocatalysis combined H_2_O_2_ coupled hydrodynamic cavitation; 0.5% Ag-TiO_2_, 200 W UV-irradiation, photocatalyst dosage= 1 g/L, pH = 3, and MO initial concentration = 15 ppm.

The detailed synergy coefficient calculations are included in Supporting Information. For comparison, a similar study conducted by Merdoud et al.,^43^ using TiO_2_ supported on glass fiber tissue reported a γ_2_ of 9.22. Furthermore, Fedorov et al.^70^ observed a synergy coefficient of 2.5 for a comparable combined process. Similarly, Thanekar et al.,^71^ reported a synergy coefficient of 2.9 for the combined use of HC and H_2_O_2_, highlighting the superior performance of the 0.5% Ag-TiO_2_ photocatalyst in the present study. To the best of our knowledge, the synergy coefficient achieved in this study represents an unprecedented efficiency in the literature, emphasizing the system’s potential for rapid and effective pollutant degradation. Lines in Figure 7 are as per eqs 2 and 3 with rate constants listed in Table 6. Table 6 summarizes the rate constant k, the degradation rate of MO, and the synergy coefficient for the combined system PC/H_2_O_2_/HC.

**Table 6:** Degradation Kinetics and Synergistic Effects of Photocatalysis with H_2_O_2_ and HC

Operative conditions	Time of degradation [min]	Effective degradation rate constant *k*_eff_ [min^–1^]	Degradation rate [%]	Synergy coefficient γ_2_ [−]
PC alone	60	0.0024	13.9	-
HC alone	60	0.0445	83.3	-
PC + 0.01% v/v H_2_O_2_/HC	03	1.9746	100	42

### 3.6 Identification of Radical Species

Figure 8 demonstrates the impact of various scavengers on the photocatalytic degradation process using 0.5% Ag-TiO_2_ as a photocatalyst. The degradation efficiencies are compared to a control system operating without any scavenger. The highest degradation efficiency, 58.8%, is observed in the absence of scavengers, indicating the optimal performance of the Ag-TiO_2_ photocatalytic system under scavenger-free conditions. This suggests that reactive species, such as hydroxyl radicals (•OH) and superoxide radicals (O_2_^–^•), are actively involved in the degradation process when they are not hindered by scavengers.^72^ The addition of isopropanol significantly reduces the degradation efficiency to 19.7%, as isopropanol, a well-known scavenger for hydroxyl radicals (•OH), causes this marked decrease, highlighting the dominant role of these radicals in the photocatalytic activity facilitated by Ag-TiO_2_. Zhou et al.,^73^ also observed that (•OH) plays a crucial role in the photocatalytic processes involving TiO_2_, further supporting these findings. Similarly, the introduction of EDTA lowers the efficiency to 27.9%. As EDTA primarily scavenges photogenerated holes (h^+^), this result emphasizes the importance of these holes in the degradation mechanism. Methanol, which acts as a scavenger for superoxide radicals (O_2_^–^•), leads to a reduction in efficiency to 31.3%. This result underscores the critical role of superoxide radicals in the degradation process. The lowest degradation efficiency, 14.5%, is observed with KI, which acts as a scavenger for both hydroxyl radicals and photogenerated holes. This result indicates that both reactive species are essential for achieving effective photocatalytic degradation using Ag-TiO_2_.

**Figure 8:**
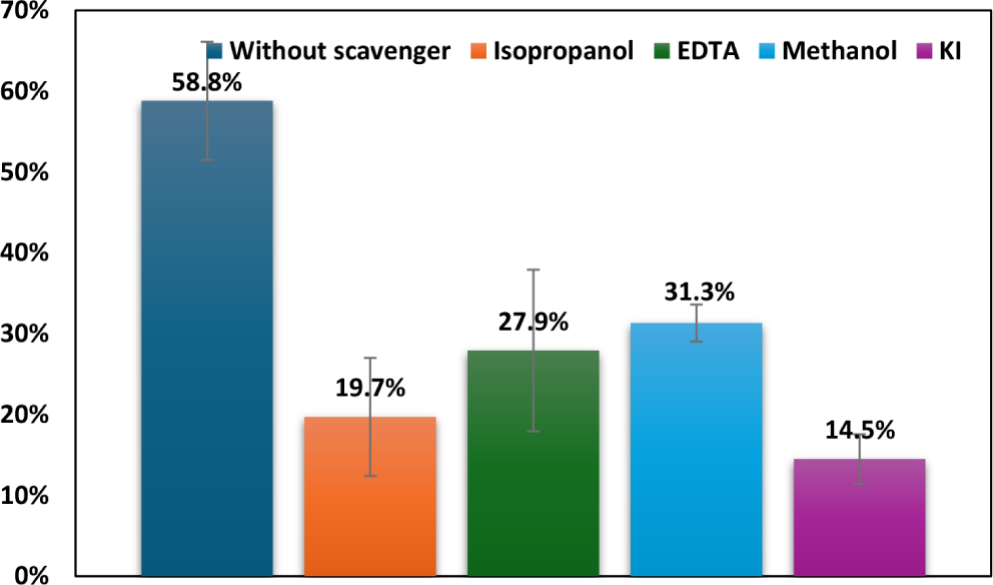
MO dye degradation with and without scavengers; 0.5% Ag-TiO_2_, 200 W UV-irradiation, photocatalyst dosage= 1 g/L, pH = 3, and MO initial concentration = 15 ppm.

### 3.7 Effect of Anions/Water Matrix

The effect of anions and the water matrix on photocatalytic degradation can significantly influence the efficiency and mechanisms of the process. Various anions, such as chloride (Cl^–^), sulfate (SO_4_^2–^), or bicarbonate (HCO_3_^–^), can either inhibit or enhance the photocatalytic activity by interacting with the active sites on the photocatalyst surface or influencing the generation of ROS. For instance, chloride ions can form complexes with metal centers or react with hydroxyl radicals, potentially reducing the availability of these radicals for pollutant degradation. In contrast, bicarbonate ions may act as scavengers of hydroxyl radicals, thereby reducing the degradation efficiency. The water matrix itself also plays a crucial role by providing a medium for the reaction, affecting parameters such as pH, ionic strength, and solubility of reactants, which can alter the charge distribution on the catalyst surface and affect the photocatalytic reaction rate. The presence of dissolved organic matter or other impurities in natural or wastewater matrices can further complicate the process, as they may either compete for active sites or form secondary products that affect the overall degradation pathway. Therefore, the interplay between anions and the water matrix must be carefully considered when evaluating the photocatalytic performance in real-world applications.

### 3.8 The Environmental Fate of Catalysts after Usage

The environmental fate of catalysts after usage is a crucial aspect of their sustainability in environmental applications. For catalysts like Ag-TiO_2_, the primary concern revolves around their potential release into the environment, especially if it is not properly immobilized. When Ag-TiO_2_ is used in wastewater treatment, the risk of leaching, particularly of silver nanoparticles, poses a threat to aquatic ecosystems due to its toxicity to aquatic organisms.^30^ The fate of the catalyst largely depends on factors like its stability, whether it is immobilized on a support material (such as glass fiber or ceramic foams) or used in a suspended form. Immobilization can minimize leaching and facilitate easy recovery and reuse, reducing the likelihood of environmental contamination. In contrast, if the catalyst is used in its dispersed form, recovery becomes more challenging and increases the potential for environmental pollution.^74^ To mitigate these concerns, strategies like efficient filtration, regeneration methods, and safe disposal of spent catalysts are essential.^75^ Additionally, advancing the development of eco-friendly synthesis techniques for catalysts can further reduce their environmental impact, ensuring they remain as sustainable as possible throughout their lifecycle.^76^

## 4 Summary and Conclusions

In this study, the degradation of MO dye solution was investigated at the laboratory scale using a combination of AOPs. The research focused on Ag-doped TiO_2_ photocatalysts, H_2_O_2_, and vortex-based HC to enhance MO degradation. The Ag-doped TiO_2_ photocatalysts were successfully synthesized via a solid-state method, and their structural and morphological properties were confirmed through various characterization techniques, including XRD, SEM-EDS, and BET surface area analyses.

The study identified 0.5% Ag-TiO_2_ as the optimal concentration for photocatalytic performance, achieving the highest degradation efficiency under both 60 W (24.6%) and 200 W (58.8%) UV lamp intensities. Additionally, the optimal photocatalyst loading was determined to be 1 g/L, which provided the best degradation results. The incorporation of 0.01% v/v H_2_O_2_ significantly enhanced the degradation efficiency, reaching 94% within 60 min and yielding a high synergy coefficient of 24.44. Furthermore, the integration of PC, H_2_O_2_, and HC led to remarkable improvements, achieving complete MO degradation in just 3 min (1.5 passes) with a high synergy coefficient of 42. This highlights the strong interaction between the AOPs, resulting in a highly efficient treatment system. The role of reactive species in the degradation mechanism was confirmed through scavenger tests using isopropanol, EDTA, methanol, and KI, which significantly reduced degradation efficiency. This underscores the contribution of hydroxyl radicals, photogenerated holes, and superoxide radicals in the reaction. Our study also highlighted the influence of anions and the water matrix on photocatalytic degradation, demonstrating how species like chloride and bicarbonate can affect reactive oxygen species generation and overall efficiency. Additionally, we addressed the environmental fate of Ag-TiO_2_ catalysts after usage, emphasizing the necessity of immobilization to mitigate silver leaching and highlighting the importance of sustainable recovery strategies to reduce environmental impact

Overall, the results of this study demonstrate that the combination of Ag-TiO_2_ with multiple AOPs, specifically H_2_O_2_ and HC, offers a highly effective strategy for accelerating MO degradation due to the strong synergy between these processes. These findings provide valuable insights for researchers exploring effluent treatment through the coupling of PC, H_2_O_2_, and HC.

## Supplementary Material



## Notes

Additional data supporting the findings of this study, including experimental setups, photocatalyst preparation details, UV spectroscopy analysis, molar ratio calculations, and synergy coefficient calculations (PDF)
