# Supplementary material for: Methyl Orange Degradation Using Ag-Doped TiO2, H2O2, and Hydrodynamic Cavitation
Source: ACS Omega. 2025 May 19;10(21):21377–90. doi: 10.1021/acsomega.5c00034 (PMC12138649; doi:10.1021/acsomega.5c00034)
Supplement: Supplementary file 1 [file ao5c00034_si_001.pdf]

Supplementary Information

Methyl Orange Degradation using Ag-Doped TiO_2, H_2O_2, and Hydrodynamic Cavitation

Ryma Merdoud[a,b], Farid Aoudjit[a], Lotfi Mouni[c], Vaishnavi Honavar[b], Roja Parvizi Moghadam[b], Manoj Palabathuni[b], Vivek V. Ranade[b,*]

[a] Laboratoire Matériaux et Développement Durable, Faculté des Sciences et Sciences Appliqués, Université de Bouira, 10000 Bouira, Algeria
[b] Department of Chemical Sciences and Bernal Institute, University of Limerick, Ireland
[c] Laboratoire de Gestion et Valorisation des Ressources Naturelles et Assurance Qualité, Faculté SNVST, Université de Bouira, 10000, Algeria

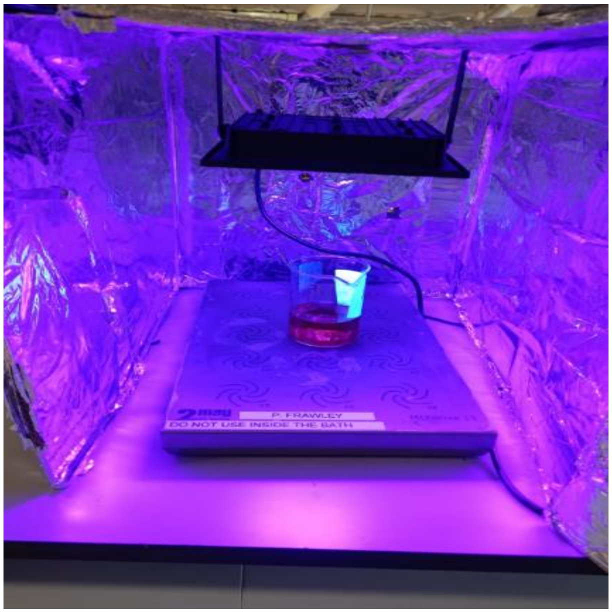

Fig.S1. Experimental setup of PC

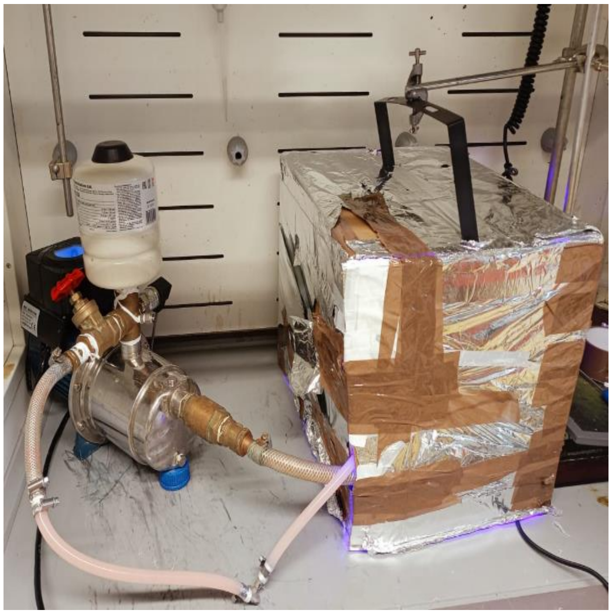

Fig.S2. Experimental setup of PC/HC

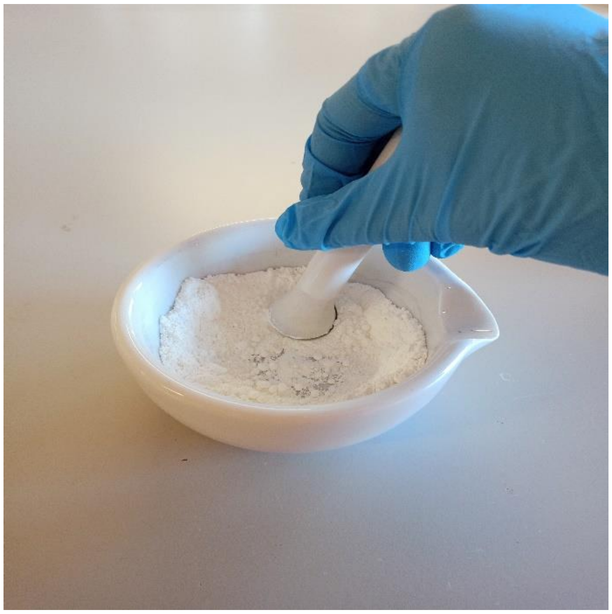

Fig.S3. Photocatalysts preparation process

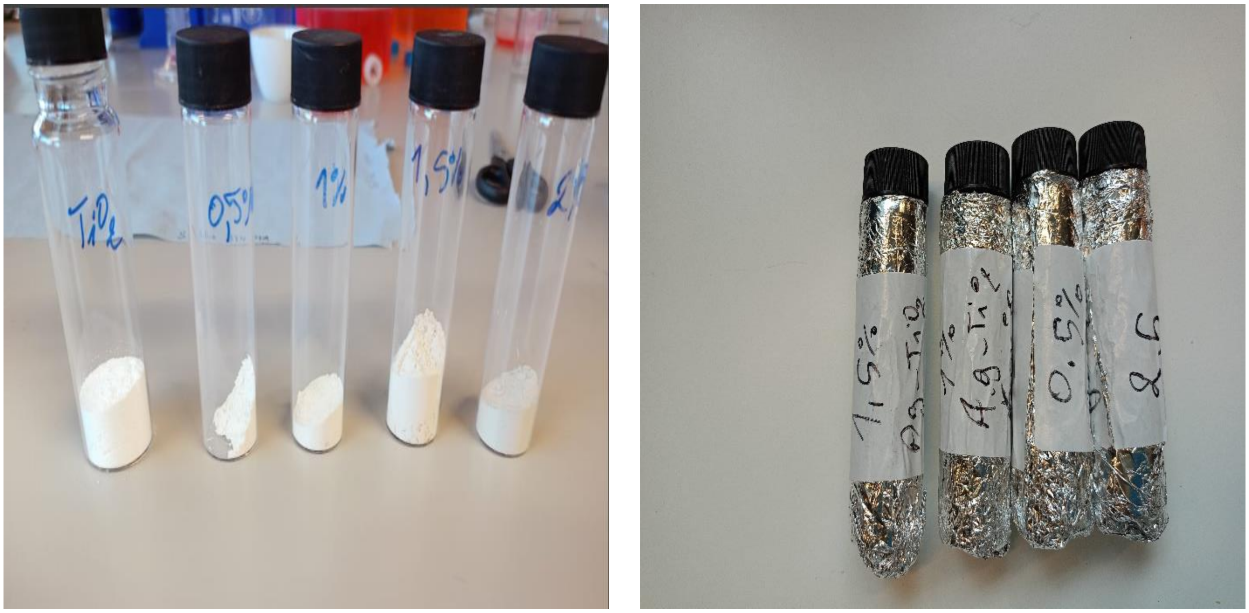

Fig.S4. Prepared Photocatalysts

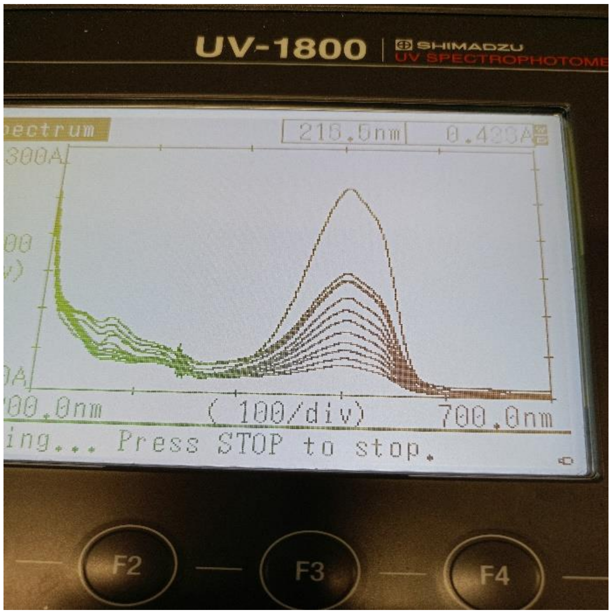

Fig.S5. UV spectroscopy analysis of MO samples using PC/0.01% v/v H_2O_2

S1. Calculation of molar ratio of oxidant to pollutant

Calculation of the moles of MO

The concentration of MO is given as 14–15 ppm, and we'll assume 14 ppm for this calculation.

1 ppm is equivalent to 1 mg/L, so:

$$C_{MO} = 14\, mg/L$$

The volume of the MO solution used is 200 mL (0.2 L). The mass of MO in the solution is:

$$m_{MO} = 14 \ \times 0.2$$

$$m_{MO} = 2.8\, mg$$

To convert this to moles, we use the molar mass of MO, which is approximately 327.33 g/mol:

$$n_{MO} = \frac{2.8 \times 10^{-3}}{327.33}$$

$$n_{MO} = 8.57 \times 10^{-6}\, mol$$

Calculation of the moles of H_2O_2

The concentration of H_2O_2 used in the experiment is given as 30% v/v, which is equivalent to 30 mL of H_2O_2 in 100 mL of solution. We'll assume a 200 mL solution of MO, so the amount of H_2O_2 used is 30% of 200 mL:

$$V_{H2O2} = 0.3 \times 200$$

$$V_{H2O2} = 60\ mL$$

The density of 30% H_2O_2 is approximately 1.11 g/mL. The mass of H_2O_2 in 60 mL is:

$$m_{H2O2} = 1.11\ \times 60$$

$$m_{H2O2} = 66.6\ g$$

The molar mass of H_2O_2 is 34.01 g/mol, so the moles of H_2O_2 are:

$$n_{H2O2} = \frac{66.6}{34.01}$$

$$n_{H2O2} = 1.96\ mol$$

Calculation of the molar ratio of H_2O_2 to MO

Now that we have the moles of MO and H_2O_2, we can calculate the molar ratio:

$$Molar\ ratio = \frac{n_{H2O2}}{n_{MO}}$$

$$Molar\ ratio = \frac{1.96}{8.57 \times 10^{-6}} = 228{,}741$$

S2. Calculation of synergy coefficient

$$\gamma_2 = \frac{k_{PC+\mathrm{H_2O_2}+\mathrm{HC}}}{k_{\mathrm{PC}} + k_{\mathrm{H2O2}} + k_{HC}}$$

The value of k_{HC} at 1.5 bar was calculated using previous work [43] as:

$$k_{eff} = \beta \Phi$$

$\beta = \frac{Q}{V}$ with Q=1.25 LPM, and V=2.5 L. The per-pass factor, Φ, was reported as 0.089. Thus,

$$k_{eff,HC} = \frac{1.25}{2.5} * 0.089$$

$$k_{eff,HC} = 0.0445$$

$$\gamma_2 = \frac{1.9746}{0.0024 + 0.0003 + 0.0445} = 41.85$$
